# Engineered exosomes in emerging cell-free therapy

**DOI:** 10.3389/fonc.2024.1382398

**Published:** 2024-03-26

**Authors:** Chaohua Si, Jianen Gao, Xu Ma

**Affiliations:** National Research Institute for Family Planning, Chinese Academy of Medical Sciences & Peking Union Medical College, Beijing, China

**Keywords:** exosome, cell-free therapy, engineered exosomes, modification, drug development

## Abstract

The discovery and use of exosomes ushered in a new era of cell-free therapy. Exosomes are a subgroup of extracellular vesicles that show great potential in disease treatment. Engineered exosomes. with their improved functions have attracted intense interests of their application in translational medicine research. However, the technology of engineering exosomes still faces many challenges which have been the great limitation for their clinical application. This review summarizes the current status of research on engineered exosomes and the difficulties encountered in recent years, with a view to providing new approaches and ideas for future exosome modification and new drug development.

## 1 Background

Following the studies of Chargaff and West in 1946, which opened the field of extracellular vesicle (EV) biology, several studies in 1990 showed that exosome expression levels were altered in disease states. Since then, research on exosomes in the field of disease treatment has grown rapidly (1–5). For example, exosomes of immune cell origin have been shown to affect the function of the immune system (6). In addition, with the development of exosome research technology, researchers have the ability to detect individual exosomes, announcing that exosome research has entered the era of individual exosomes (7, 8).

Exosomes, with an average diameter of ~100 nanometers, are a subset of EVs (9). Almost all types of cells release exosomes, which can be seen as a regular physiological activity of cells (10). Cells are the most basic building blocks of the human body, and their abnormal state often leads to disease. With the development of research methods and techniques, researchers have discovered that in addition to cells, exosomes also play a crucial role in the onset and progression of disease (9, 11, 12). Exosomes are usually characterized by low immunogenicity, high safety, high tissue penetration, and can circulate to almost all body cavities (13). In addition, exosomes secreted by different cells have different tissue selectivity (14).

With the deepening of exosome research, the great potential of engineered exosomes in the treatment of diseases, especially cancer, has been gradually recognized. Currently, engineered exosomes are mainly used to enhance the therapeutic effect of diseases by enhancing targeting, regulating gene expression, acting as drug carriers, altering the tumor microenvironment, and regulating inclusion bodies, etc. However, there is no one technique for all situations. Researchers must endow exosomes with a variety of characteristics depending on the actual need. For example, melanoma-derived, loaded doxorubicin (DOX) and peptide-targeted exosomes preferentially target disease sites while minimizing systemic off-target problems (15, 16).

This paper reviews the development process of exosomes in recent years, the application of immune cell-derived exosomes in tumor therapy and the difficulties encountered in the clinical translation of engineered exosomes. In order to provide a new method and idea for the clinical translation of clinically applied exosomes.

## 2 The biogenesis and isolation of exosomes

**Figure 1:**
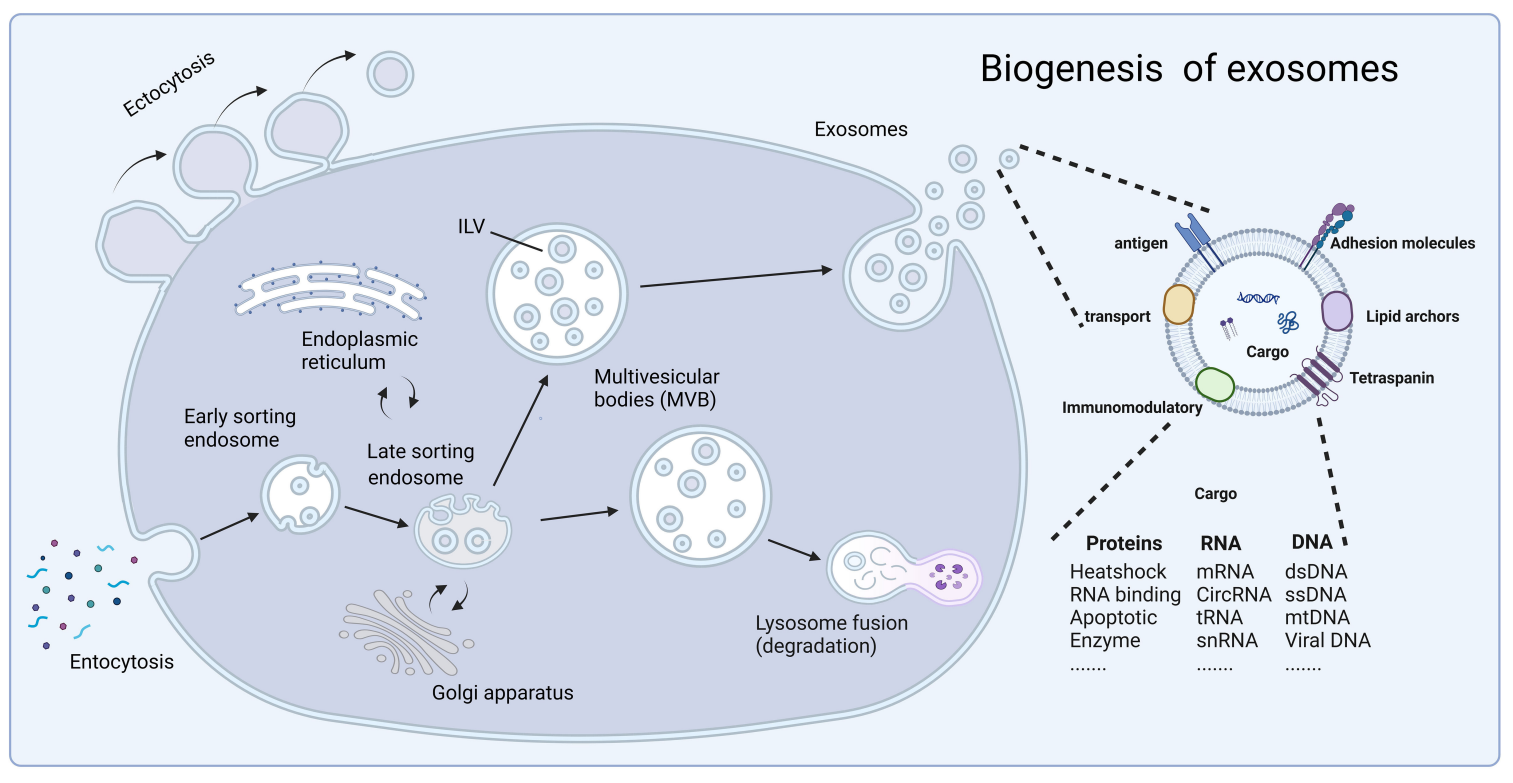
The biogenesis of exosomes. Fluid and extracellular components enter the cell by endocytosis, and exosome production is divided into two steps: plasma membrane double invagination and intracellular multivesicular body formation. In this, the trans-Golgi network and endoplasmic reticulum play a facilitating role. Subsequently, exosomes are released through cytolysis with lipid bilayers oriented similarly to the plasma membrane. Exosomes contain different types of cell surface proteins, intracellular proteins, RNA, DNA, amino acids, and other metabolites, and serve as mediators of proximal and distal intercellular communication in health and disease. At the same time, secretion and uptake of exosomes accomplishes the exchange of substances between cells and the transfer of cellular information from donor cells to recipient cells. Created with BioRender.com.

Exosome production is divided into two main steps, plasma membrane double invagination and intracellular multivesicular body (MVB) formation (10). Specifically, the plasma membrane invaginates to form a cup-shaped structure, leading to the re-formation of early sorting endosomes (ESEs). During synthesis, the trans-Golgi network and endoplasmic reticulum play a facilitating role (17–22). After a period of time, the ESE matures into late sorting endosomes (LSEs), which eventually form MVBs. MVBs are formed by double invagination of the plasma membrane, resulting in the formation of MVBs containing multiple intraluminal vesicles (ILVs). During release, MVBs are degraded by fusion mainly with lysosomes or autophagosomes or by direct fusion with plasma membranes to release exosomes (18, 23). At the same time, exosome uptake is an important step in intercellular cargo transportation. The uptake of exosomes by recipient cells occurs mainly through endocytosis, phagocytosis, or direct fusion with the plasma membrane (24–26). Exocytosis can be categorized into lattice protein-mediated exocytosis, lipid raft-mediated exocytosis, and heparan sulfate proteoglycan-dependent exocytosis. Ultimately, successful uptake of exosomes accomplishes the exchange of intercellular substances and transfers cellular information from the donor cell to the recipient cell (**Figure 1**).

The isolation of exosomes is important for the study of their mechanisms and clinical applications, and the production of large-scale, high-purity and low-cost exosomes is a major challenge that limits the use of exosomes for clinical translation. Currently, the main methods for exosome isolation include ultracentrifugation, ultrafiltration, chromatography and precipitation (27). In recent years, researchers are continuously improving exosome extraction methods in order to obtain higher quantity and quality of exosomes. For example, the immunoaffinity method originally utilized protein interactions to extract exosomes. To improve this method, researchers have used submicron-sized magnetic particles for immunoaffinity capture-magnetic immunocapture, which increases the amount of exosomes captured by 10-15 times (28). Another example is the application of microfluidics to the isolation, detection and analysis of exosomes. Microfluidics-based separation techniques not only utilize common separation elements such as size and density, but also incorporate some innovative sorting devices such as electrophoresis and electromagnetism (29). The researchers utilized a micro- and nanofluidic device to separate and capture exosomes of liposarcoma origin, increasing the throughput per unit of time by a factor of five (30). While this improvement has led to improved yields of exosomes and is achievable in the laboratory, they are often limited in clinical-grade applications by various constraints, such as high costs, low yields, and complex procedures.

In conclusion, isolating high-quality exosomes is a crucial step in studying their effects in tumor therapy, and the usual methods often fail to meet the multiple requirements for isolation, such as high purity, high throughput, low cost, and few volume constraints. Therefore, integration of multiple methods is essential for isolating exosomes with high purity and yield, which is also an important direction for the future development of engineered exosomes.

## 3 Roles and functions of exosomes *in vivo*

**Figure 2:**
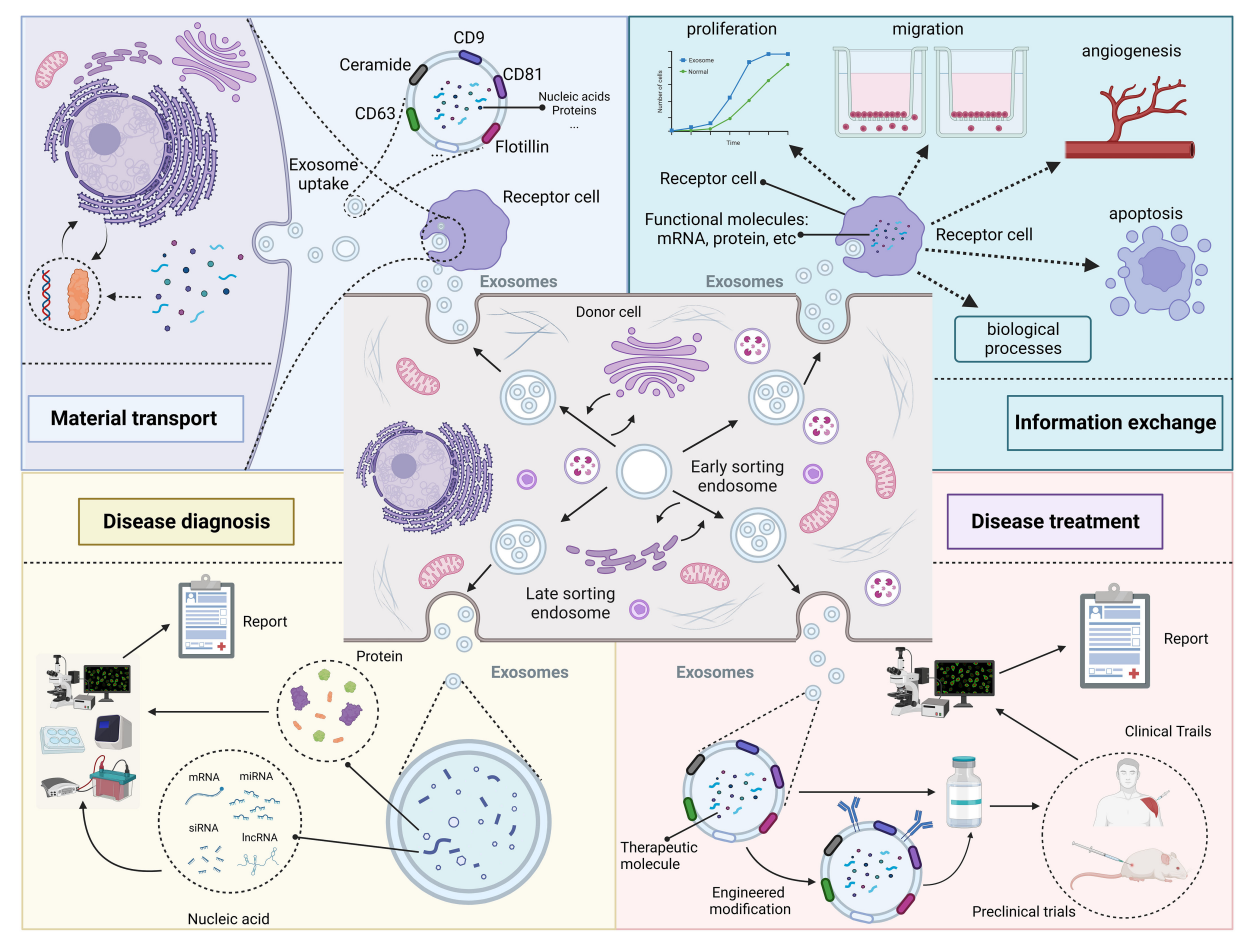
Roles and functions of exosomes *in vivo*. Exosomes from different cellular sources contain different nucleic acid and protein components and perform different functions. Some exosomes secreted by donor cells contain substances essential for the survival of recipient cells, which play a role in substance transportation. In addition, exosomes carry genetic information that can enter the recipient cell and cause changes in its phenotype. Changes in exosome composition often reflect the health status of the organism and can be used for disease diagnosis. Exosomes contain specific therapeutic molecules, such as miRNA, which can be used in disease treatment. Created with BioRender.com.

When exosomes were discovered in the early days, researchers usually thought they were just metabolic waste products of cells (31). However, with the improvement of research techniques, researchers are coming to realize that exosomes may play an important role in the biological process of disease (10). This review summarize the role of exosomes in disease from four perspectives: material transport, information exchange, disease diagnosis and disease treatment (**Figure 2**).

### 3.1 Material transport

In general, when an exosome secreted by one cell enters another cell, it brings a variety of active substances into the recipient cell, realizing the transportation of substances between different cells. Many studies have demonstrated that intercellular substance exchange is crucial for intracellular substance homeostasis. For example, mRNAs are essential for protein production in cells, and it has been found that there is a class of mRNAs specifically present in exosomes, which can accompany the exosomes into the recipient cells, translate and alter their protein expression (32, 33). In addition, some proteins undergo post-translational lipid modification, which prevents them from diffusing freely in the hydrophilic extracellular environment, making it difficult to transport substances between cells, as in the case of WNT. Exosomes, on the other hand, can transport proteins directly into recipient cells for substance replenishment (34). Indeed, exosomes have emerged as effective vehicles for the diffusion of lipophilic ligands in the extracellular environment (35–38). At the same time, uptake and efflux of exosomes are essential for the renewal of cell membrane components.

### 3.2 Information exchange

Recent studies have confirmed that exosomes can be transported over long distances *in vivo*, mediate intercellular information transfer, and affect various physiological functions of recipient cells, especially the nucleic acids and proteins contained in exosomes (39, 40). The message-exchange function of exosomes has been demonstrated in a variety of diseases, particularly cancer. For example, genetic experiments conducted by researchers in mice have shown that small amounts of functional mRNA can follow exosomes into receptor cells to act, and that the probability of this occurring is increased in mouse models with peritonitis or subcutaneous tumors (41–43). In addition to nucleic acids, exosomal proteins reflect the protein composition of donor cells, and exosomal proteins also cause phenotypic changes in recipient cells after uptake by recipient cells. In addition, exosomes secreted by glioblastoma cells that highly express epidermal growth factor receptor variant III (EGFRvIII) are specifically enriched for migration-promoting protein molecules and enhance their migratory ability after being taken up by recipient cells. Second, neural stem cells affected by inflammatory factors produce exosomes containing interferon gamma. When ingested by receptor cells, it induces the production of interferon γ by receptor cells (44). The above results suggest that cell-generated signaling molecules can be loaded into exosomes and selectively induce specific signals in recipient cells to regulate various biological processes.

### 3.3 Disease diagnosis

Exosomes contain membrane proteins, cytoplasmic and nuclear proteins, extracellular matrix proteins, metabolites and nucleic acids (45–48). Meanwhile, exosomes are heterogeneous; exosomes obtained from different cell sources, isolation methods and isolation stages have different sizes, components and functions (20, 49, 50). Exosomes from different sources may have different effects on the same cell, which may be due to differences in the inclusions of the exosomes. Also, exosomes from the same cellular source may have different effects on different receptor cells, and this heterogeneity may be due to the role and function of the receptor cells (51). Based on these reasons, the membrane proteins of exosomes, or the nucleic acids and proteins contained in exosomes, can be used as biomarkers of disease, especially in the diagnosis of cancer. In cancer, in addition to the ease of obtaining samples compared to other assays, exosomes have the significant advantage that only living cells can release exosomes, and the contents of tumor cells reveal information about the living cells of the tumor, which is more conducive to the patient’s diagnosis of the disease (52–55). In addition to their potential as diagnostic markers, exosomes can improve the sensitivity of other methods such as liquid biopsy (52). The combination of exosomal RNA and ctDNA produced a significant increase in the number of mutant copies compared to ctDNA alone, significantly improving the chances of detecting mutations from blood samples (52). Thus, the combined analysis significantly improved the correlation of biomarkers with treatment outcomes compared with ctDNA alone, and the method significantly improved the success rate of liquid biopsy trials (53, 54).

### 3.4 Disease treatment

Exosomes have shown great potential in the treatment of a wide range of diseases, and cell-free therapies represented by exosomes have greatly expanded the therapeutic approaches for a wide range of diseases, including cancer (56–59). First, compared with other drug delivery vehicles, exosomes as cell products have extremely low immunogenicity (60–62). For example, in triple-negative breast cancer, exosomes with effective lung-targeting ability were identified from autologous breast cancer cells, and exosomes were used to deliver siRNAs to improve drug delivery to pre-metastatic niche (PMN) in the lung. Demonstrating favorable biocompatibility, higher lung affinity and gene silencing effects, it is a promising strategy for suppressing postoperative breast cancer metastasis (63). In addition, exosomes have the ability to cross the blood-brain barrier and can circulate in the body for longer periods of time to maintain therapeutic effects (64). The ability to deliver drugs efficiently is one of the most important factors affecting the efficacy of glioblastoma (GBM), a major obstacle when the blood-brain barrier exists. Wang et al. prepared a biomimetic nanodrug delivery platform using exosomes to efficiently target the brain without target modification, and delivered drug and immune adjuvants at the same time for safe and efficient chemo- and immuno-therapy of GBM (65). Currently, cancer is one of the most important diseases facing mankind and one of the major causes of human deaths worldwide (66). Tumors are treated in a variety of ways, including surgery, radiotherapy, chemotherapy, immunotherapy, and targeted therapy. Radiotherapy and chemotherapy are very common treatments, but many patients have lower treatment effects and poorer prognosis (67, 68). Drug resistance in tumor cells is one of the underlying causes (69). It has been reported that Mesenchymal stem cell-derived (MSC-derived) exosomes can directly deliver functional proteins and RNAs, such as miRNAs, which in turn modulate apoptosis-associated proteins and reduce cellular chemotherapy resistance (70). Exosomes from immune cells can also directly kill tumor cells. For example, the study by Li et al. proved that NK cells are known to exert cytotoxicity by cleaving cytotoxic substances in granules, and the transmembrane protein Fasl on their surface determines the fate of target cells, which provides a new approach to the treatment of secondary hepatocellular carcinoma. Compared to NK cells, exosomes from NK cells are enriched to more Fasl and perforin proteins, and have a greater ability to kill tumor cells (71). Exosomes also play an important role in the remodeling of the tumor microenvironment, where tumor cells live. For example, macrophages are abundant in the TME and have two distinct phenotypes, including m1-polarized macrophages and m2-polarized macrophages. m1-polarized macrophages and their exosomes kill tumor cells by promoting immune responses in the tumor microenvironment (72). In addition to the above pathways, cellular exosomes are rich in proteins and nucleic acids (e.g., circRNAs, miRNAs, and lncRNAs), which play an important role in the malignant phenotype of tumors. In conclusion, these studies demonstrate the important function of exosomes in tumor therapy (73–75).

However, although exosomes have shown great potential in the treatment of a wide range of diseases, there are still some difficulties that need to be addressed. For example, the heterogeneity of cell growth and proliferation can lead to a decrease in exosome function, thus affecting the therapeutic effect after drug preparation. Second, exosomes are secreted by different cells in different microenvironments as a means of exchanging material and information between cells and between cells and the environment. Thus, the composition and production of cellular exosomes are to some extent related to the cellular state and cell culture conditions. Different cell culture medium compositions and oxygen levels in cell culture led to differences in the production and composition of exosomes, which in turn lead to differences in their function. Third, the composition and function of exosomes produced by different cells vary widely. The packaging mechanisms of effector molecules in exosomes of different cell types are not clear, and the existence of packaging signals that influence the entry of molecules into exosomes remains to be explored. Fourthly, the development of quality standards and the selection of quality control methods in the production process of exosomes still need to be explored, and the efficient and stable long-term storage of exosomes is also an important research element in the study of exosome chemogenesis. Engineering strategies for exosomes, such as targeted modifications, provide a new approach and idea to address these issues. In addition, engineered exosomes in clinical translation is also faced with the source, regulation and cost of many aspects of the problem, how to solve these problems will be an important direction for the future development of engineered exosomes.

## 4 Outstanding properties of engineered exosomes

**Figure 3:**
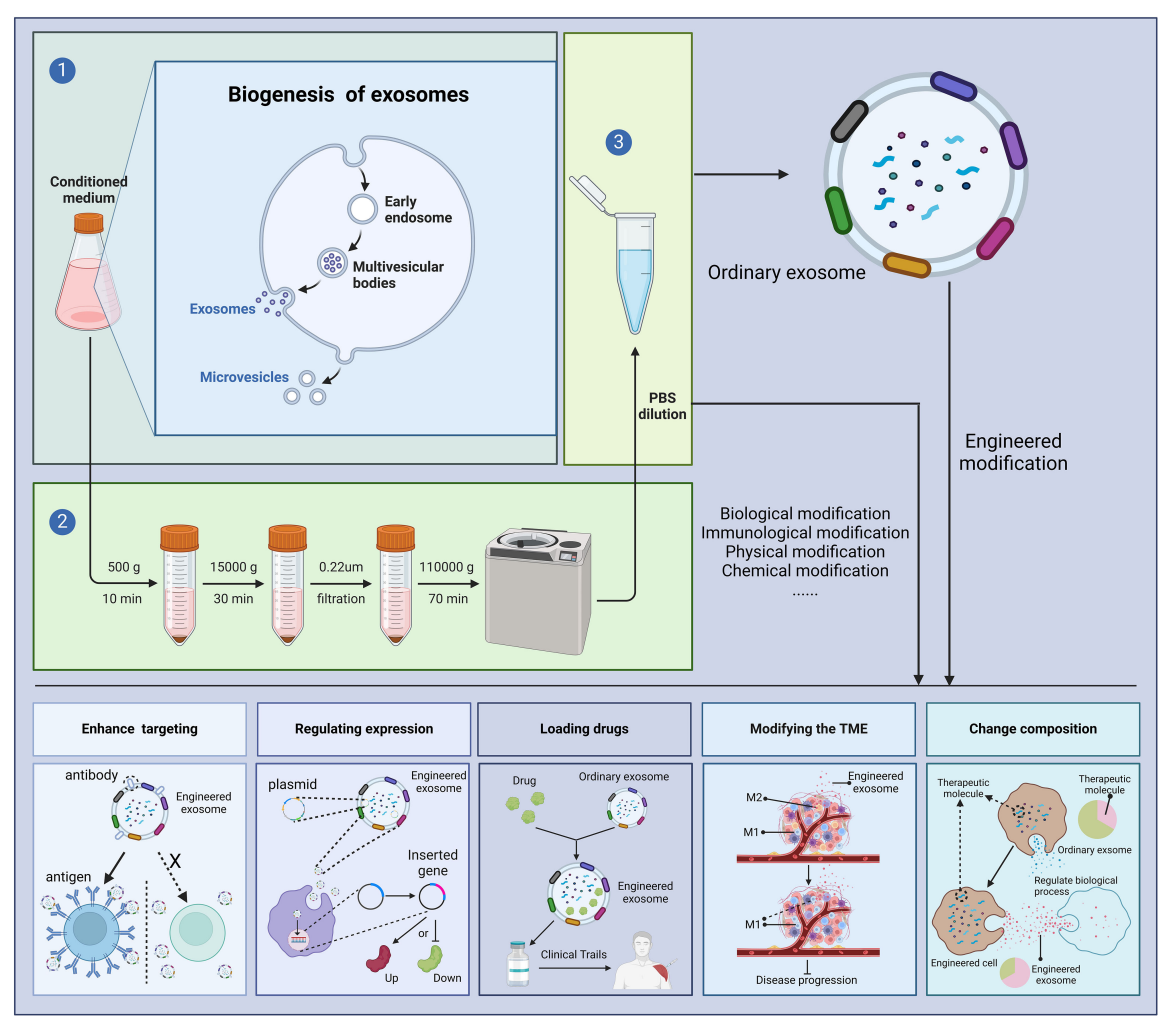
Outstanding properties of engineered exosomes. Ultracentrifugation is a very common method for exosome isolation. Depending on the purpose of the experiment, we can modify the donor cells or exosomes, including biological modification, immunological modification, physical modification and chemical modification, to enhance the various potentials of exosomes and to treat diseases. "X" means that in the absence of a targeting peptide, there is no targeting of the exosome into the receptor cell. Created with BioRender.com.

Above, we have discussed the role of exosomes in the treatment of diseases. At the same time, exosomes can be engineered, and engineered exosomes can enhance or even give some new properties to exosomes (76). There are many studies using engineered exosomes to treat diseases (77–79). Currently, the engineering of exosomes is divided into four main routes: biological modification, immune modification, physical modification and chemical modification. The most common method for extracting exosomes is ultrafast centrifugation. In this paper, we will introduce the advantages of engineered exosomes over natural exosomes and their applications in various disease models (**Figure 3**).

### 4.1 Enhance exosome targeting

It has been found that exosomes can enter receptor cells through a variety of pathways. It can either fuse directly with the plasma membrane or be taken up by the recipient cell through phagocytosis and endocytosis mediated by vesicular and lattice proteins (20, 80). From the current research, it is clear that exosomes can enter almost any cell. One of the fundamental reasons that exosomes are internalized by cells is the ability of these receptor cells to recognize exosome membrane surface molecules. For example, the CXCR4/SDF-1α interaction has been shown to mediate the selective transfer of endothelial colony-forming cell-derived exosomes to renal (81). These receptor-ligand interactions enhance exosome-targeted delivery (82). This mechanism of exocytosis suggests that we can design exosome membrane surface proteins to facilitate their targeted transport capacity (76). In recent years, researchers have identified a number of specific proteins on the membrane surface of exosomes, such as lysosome-associated membrane protein 2b (Lamp-2b), tetramin (CD63, CD81, CD9), lactomucin (LA), and glycosylphosphatidylinositol (GPI). In addition, surface modification strategies of exosomes, such as genetic engineering and covalent and noncovalent modification of exosomes, have been used for exosome-targeted therapy (83–87). For example, Lam2b, a commonly used membrane surface protein, is significantly enriched in dendritic cell-derived exosomes (88, 89). Lamp2b is highly expressed on the cell surface and is frequently fused to target peptides to obtain peptide-modified exosomes for disease therapy (90). Meanwhile, the use of plasmids encoding target ligands genetically engineered to fuse with transmembrane proteins in exosome donor cells has been widely used to create engineered exosomes (83). For example, Kim et al. co-transfected HEK293 cells with pcDNA - cardiac-targeting peptide (CTP)-Lamp2b to generate cardiac-targeting exosomes (91). Regardless of the modification, the ultimate goal is to allow the exosome to enter as many receptor cells as possible, rather than other cells, to improve therapeutic efficacy and minimize side effects.

### 4.2 Regulating gene expression

Many human diseases have a genetic basis, and the timely detection and testing of these genes is important for the diagnosis and treatment of diseases (92). Studies on exosomes have shown that their enriched nucleic acids play a crucial role in regulating gene expression in recipient cells (74, 75, 93). In addition, exosomes can serve as vectors for the regulation of gene expression. A common approach to treating hereditary diseases is gene therapy, in which genetic material is introduced to treat the disease (94, 95). The choice of vector significantly affects the efficacy of gene therapy. Adeno-associated virus (AAV) is widely used in gene therapy. However, AAV vectors often elicit an immune response in the host, leading to their rapid degradation, which seriously affects the effectiveness of gene therapy (96). Cell-secreted exosomes are highly biocompatible, low clearance, and targeted delivery, making them well suited for gene delivery (97). Encapsulation of exosomes protects AAV vectors from host cells and delivers them through the cytoplasmic membrane (98). Engineered exosomes can also be used directly as gene expression regulatory vectors (99). For example, researchers have designed an exosome-based chondrocyte-targeted miRNA delivery system for cartilage defect repair (100). Specialized exosomes loaded with the CRISPR-Cas9 system can be accurately delivered to target cells (101, 102). The lipid bilayer of the exosome membrane fuses with liposomes to form exosome-liposome heterodimers that can encapsulate and deliver large molecules of DNA (103, 104). Therefore, exosomes have great value and translational potential as gene expression regulatory vectors.

### 4.3 As a tool of loading drugs

In addition to serving as gene expression regulatory vectors, exosomes can also be used to deliver drug (105). Prior to the discovery of exosomes, lipid nanoparticles (LNPs) have been recognized as advantageous carriers for the protection, transport and delivery of various drugs and vaccines to cells (106). However, the low bioavailability, toxicity and immune response of LNPs complexes still constrain their clinical application (105). After the discovery of exosomes, researchers realized that exosomes have many of the properties of liposomes that give them an advantage in drug delivery, such as the ability of exosomes to cross biological barriers, low immunogenicity, and the presence of unique targeting proteins on the membrane (107, 108). Take macrophage-derived exosomes as an example. Macrophage-derived exosomes have surface membrane properties similar to those of macrophages, and thus M1 - macrophage-derived exosomes (M1-exos) can be used to deliver a variety of anticancer drugs for tumor therapy (109). Harney et al. developed M1-exo/PTX and M1-exo/DOX for solid tumor mice (110). Kim et al. use ultrasound to load PTX into M1-exos to treat drug-resistant tumors (111). Nie et al. demonstrated that M1-exo blocked CD47 and SIRPα and converted M2 macrophages to M1 macrophages, thereby enhancing macrophage phagocytosis of tumor cells (112). In addition to macrophages, exosomes from a variety of cells have been explored for the treatment of diseases, including cancer, potentially offering a new cell-free therapy.

### 4.4 Modifying the tumor microenvironment

The inhibitory tumor microenvironment severely affects the effectiveness of immunotherapy, and researchers have found that engineered exosomes can remodel the tumor microenvironment to improve the effectiveness of immunotherapy. For example, M1-type macrophages are usually considered as tumor-killing macrophages, which mainly play anti-tumor and immune-promoting roles. M2-type tumor-associated macrophages are a major subpopulation of suppressor immune cells. How to reprogram macrophages from M2-type to M1-type is an important way to reverse the immunosuppressive microenvironment of tumors (113, 114). M1 macrophage-derived exosomes loaded with antisense oligonucleotides (ASOs) targeting STAT6 induce the expression of nitric oxide synthase 2 (NOS2), an M1 macrophage marker that leads to the remodeling of the tumor-immunosuppressive microenvironment and the activation of CD8 t-cell-mediated adaptive immune responses (115). In addition to macrophages, fibroblast activating protein alpha (FAP) cancer-associated fibroblasts (CAF) are important targets for remodeling the tumor microenvironment. targeting of FAP genes to tumor-derived exosome-like nanovesicles (eNVs-FAP) triggers a potent and specific cytotoxic T-lymphocyte (CTL) immune response against both tumor cells and FAP CAFs in multiple models of Remodeling of immunosuppressive TME (116).

### 4.5 Increase therapeutic component

The role of natural exosomes in tumor therapy relies on the substances they contain, such as nucleic acids and proteins (117, 118). For these reasons, researchers have attempted to enhance the therapeutic capabilities of exosomes by altering their composition. Exosomes contain a variety of non-coding rna, especially miRNAs, which are very important exosomal cargoes that can influence the expression of various oncogenes and tumor suppressors, thus affecting the course of the disease (32, 39, 119, 120). The researchers found that insertion or deletion of these specific cellular motifs or exonic motifs in the miRNA increased or decreased the amount of the corresponding mirna in the intracellular production of exosomes. In addition, increased miRNA delivery mediated by EXOmotifs resulted in enhanced repression of target genes in distal cells (121). Therefore, improving the efficacy of exosome therapy by altering exosome composition may be a new direction.

## 5 Engineered exosomes can be used as drug delivery platforms for disease therapy

### 5.1 Exosomes as nanodelivery systems

The characterization of exosomes and the emergence of exosome engineering strategies have greatly expanded their capabilities as nanodelivery systems. First, a variety of modification strategies can enhance or confer certain properties to exosomes, and common modification strategies include biological, chemical, physical, and immunological modifications. One example regarding biomodification is the enhancement of exosome targeting by the addition of targeting peptides. For example, in a study by Shao et al. on osteosarcoma, researchers coupled Exo-MEG3 with a tumor-targeting cRGD peptide, which demonstrated precise tumor-targeting ability and enhanced anti-tumor effects in an osteosarcoma model (122). Exosomes can also be chemically modified, an example of which is the development of a dual stimulus responsive acoustic sensitizer using exosomes by Cao et al. That is, indocyanine green (ICG), which functions as both an acoustic sensitizer and a photoacoustic (PA) visualizer, was loaded into EVs along with paclitaxel (PTX) and sodium bicarbonate (SBC) to achieve combined chemoacoustic-dynamic therapy (123). Exosomes also allow for more precise tumor targeting under conditions of external physical interference. For example, Zhang et al. modified exosomes derived from neutrophils with superparamagnetic iron oxide nanoparticles (SPIONs), allowing these nanoparticles to selectively accumulate at tumor sites under the interference of external magnetic fields (124). In addition, immunomodified exosomes can elicit strong immune responses. One example is the use of exosomes with fibroblast activating protein-alpha (FAP) as a tumor vaccine, which can elicit a strong immune response leading to tumor therapy (116). In summary, engineered exosomes have been shown to transport specific substances to specific sites at specific spaces and times, which is expected to open up new areas for future drug delivery platforms.

### 5.2 Application of engineered exosomes in disease therapy

Disease treatment methods include general treatment, drug treatment, surgical treatment, radiation therapy and so on. With the deepening of exosome research, cell-free therapy has gradually entered people’s vision as a new disease treatment method (125). Cell-derived exosomes exhibit multiple biological activities and therapeutic potential in a wide range of diseases (125). To further enhance the efficacy of cell-free therapy, researchers have begun to modify natural exosomes, and research on engineered exosomes has addressed a wide range of diseases, including tumors (100, 122, 126). For example, in cell therapy for chronic wound healing, its effectiveness is greatly limited by immune rejection and difficulties in maintaining cellular activity (127). Exosomes can provide a therapeutic effect similar to that of promoting cell regeneration and are thought to be a way to overcome these obstacles (128). Mei et al. encapsulated humscs-derived exosomes in a bioactive scaffold composed of polyvinyl alcohol (PVA)/sodium alginate (Alg) nanohydrogel (exo@H) for wound healing in diabetic rats (127). In the treatment of osteoarthritis, drug delivery to chondrocytes through the dense, non-vascularized extracellular matrix of chondrocytes is still a challenge, and there is currently no effective treatment method (129). Due to the high permeability of exosomes and their potential as vectors, He et al. have designed an exosome-based vector to achieve specific targeted delivery of miR-140 to chondrocytes in a rat model for the treatment of osteoarthritis (85, 130, 131).

### 5.3 Combination of engineered exosomes and tumor therapies

Engineered exosomes can be used in combination with other therapies in addition to modifying themselves to fight tumors. First, engineered exosomes are used in conjunction with chemotherapy drugs. For example, in the treatment of ovarian cancer, researchers used HEK-293T cells and tumor-derived exosomes loaded with PTX, which not only inhibited tumor growth but also prevented breast cancer recurrence and metastasis (123). An exosome obtained from macrophages, in combination with aminoethyl anisidine-polyethylene glycol (AA-PEG) and PTX, showed significant anticancer effects (132). Among them, PEG reduced the recognition and internalization of MPS and significantly increased the residence time of exosomes *in vivo (* 133). Second, engineered exosomes can be used in combination with radiotherapy. One example is the use of M1 macrophage-derived engineered exosomes in combination with radiotherapy to treat lung cancer by Ma et al. The mechanism is that the expression of catalase in the membrane of M1 macrophage-derived exosomes not only improves hypoxia in the tumor microenvironment, but also enhances DNA damage in tumor cells, and DNA damage repair inhibitors encapsulated in M1Exos can significantly limit DNA damage repair (134). In addition, engineered exosomes can be used in combination with gene therapy. Gene therapy involves the delivery of genetic material to a patient to treat a disease through the expression of therapeutic genes. One example is that Bose et al. encapsulated miRNA into uPA-engineered exosomes, which not only enhanced tumor-targeting ability, but also greatly improved progression-free survival of patients (135). Another example is the loading of CRISPR/Cas9 into engineered exosomes. Liu et al. loaded Cas9 RNPs into purified exosomes isolated from hepatic stellate cells by electroporation, which showed strong therapeutic potential in mouse models of acute liver injury, chronic liver fibrosis and hepatocellular carcinoma (136). In addition to the above therapies, engineered exosomes can be used in combination with photothermal and photodynamic therapies to treat tumors and have demonstrated even more powerful results, and we summarize the applications of engineered exosomes in the treatment of various diseases in recent years (**Table 1**).

**Table 1:** Engineered exosomes for cancer therapy.

Cancer	Current clinical treatment methods	Mechanism of engineered exosomes	Ref.
Breast cancer	Chemotherapy	Increase uptake of IGG by recipient cells and release PTX for disease treatment	(123)
	Chemotherapy	Increase CD8+ T cell level and serum cytokine concentration, activate GNR-mediated thermal ablation	(137)
	Chemotherapy	Blocking the function of miR-21 and attenuating DOX resistance	(138)
	Photothermal therapy	Inhibition of malignant phenotype and accelerated drug release	(139)
	Sonodynamic therapy	Trigger sonotoxicity against cancer cells	(140)
	Gene therapy	Deliver TPD52 siRNA	(141)
	Gene therapy	Deliver miRNA	(142)
	Gene therapy	Overexpression of miRNA	(135)
	Gene therapy	Block HER2 synthesis	(143)
	Immune therapy	Redirect and activate T cells	(144)
	Immune therapy	Enhanced antigen-antibody response	(145)
	Immune therapy	Induce ICD in breast cancer	(146)
	Immune therapy	Activation of cytotoxic T cells	(147)
Colorectal cancer	Chemotherapy	The malignant phenotype and drug resistance were changed, while the expression of PTEN and hMSH2 was increased	(148)
	Chemotherapy	Changes in phenotype and drug resistance, and the expression of PAEP was down-regulated. NME1 expression was up-regulated	(149)
	Immune therapy	Silence STAT6 expression and remodel the TME	(115)
Chronic myelogenous leukemia	Immune therapy	Activated T cell	(150)
Glioblastoma	Sonodynamic therapy	Enhance the ability to target and penetrate the blood-brain barrier and alter the tumor microenvironment	(151)
	Radiotherapy	Enhance the targeting efficiency of RGD-EV	(152)
	Gene therapy	Deliver miRNA degradation functional molecules	(153)
	Gene therapy	Inhibit GC progression through sponging miR-1307-3p	(154)
Hepatocellular Carcinoma	Radiotherapy	Facilitate radioiodine uptake	(155)
	Radiotherapy	The expression of miRNA was up-regulated and the phenotype of the receptor cells was changed	(156)
	Gene therapy	Delivery gene expression regulatory system	(157)
	Gene therapy	Overexpression of miRNA in recipient cells leads to phenotypic changes	(158)
Lung cancer	Radiotherapy	Macrophages were induced to activate to M1 type and immunosuppression was lifted	(134)
	Gene therapy	Decrease β-catenin expression and proliferation	(159)
	Gene therapy	Delivery gene expression regulatory system	(160)
Melanoma	Immune therapy	Enhance tumor antigen presentation capacity	(161)
	Immune therapy	Activate endogenous T cells	(162)
	Immune therapy	Activate Th1 cell responses	(163)
Non Small Cell Lung Cancer	Chemotherapy	Enhance exosome targeting	(132)
	Chemotherapy	Loaded drug	(164)
Osteosarcoma	Gene therapy	Enhance the internalization of miRNA in tumor cells and affect the phenotype of tumor cells	(165)
	Gene therapy	Deliver miRNA	(166)
Pancreatic cancer	Photodynamic therapy	Promote DCs cell maturation and produce TAA	(167)
	Gene therapy	Deliver siRNA	(168)
	Gene therapy	Enhancing drug endocytosis	(168)

## 6 Immune cell-derived engineered exosomes and tumor therapy

A variety of cells in the body can secrete exosomes, which tend to have similar properties to their secreting cells, such as anti-cancer and anti-aging (169). In recent years, numerous immunotherapies have shown great potential in cancer treatment and are gradually gaining recognition among researchers (170). Immunotherapy refers to the use of the self-protection ability of the body’s immune system to achieve the effect of killing tumors. This therapy targets human immune cells, not tumor cells, and does not cause great harm to the patient’s body as radiotherapy does, which is highly expected in terms of accuracy, effectiveness and safety (171). However, problems such as short survival time and duration of immune cells and difficulty in breaking through the solid tumor microenvironment constrain the effectiveness of immunotherapy (172). Exosomes based on immune cell sources are emerging as a potential therapeutic approach to address these limitations of cell-based therapies. Exosomes derived from immune cells have some or all of the anti-tumor properties of immune cells, as well as properties such as high penetration capacity (173). Therefore, cell-free therapy is an important complement to current anti-tumor therapies. The engineering technology of immune cell-derived exosomes confers better anti-tumor properties.

### 6.1 T cell

T cell-derived exosomes are produced only after T cells are activated. Interactions between tetraspanins, myelin, lymphocyte proteins, and ceramides were found to be critical for the biogenesis of T cell-derived exosomes (174). The study by Wang et al. proved that T-cell-derived exosomes may reflect the immune properties of T cells, for example, killing target cells directly, acting in association with B cells, producing cytokines, and creating optimal conditions for immune cells to function in a paracrine or autocrine form (175). Currently, engineering for T-lymphocytes is mainly focused on acting as gene expression vectors, which means constructing himeric antigen receptor T (CAR-T) cells for tumor therapy (176). Zhu et al. proved that CAR-T cell-derived exosomes can reduce the cytotoxicity of CAR-T therapies and have the ability to cross the blood-brain barrier and carry many cytotoxic molecules (FasL, Apo2L, perforin, etc.), which have demonstrated great efficacy as a gene-expression-regulating vector in tumor therapy (177). Hong et al.demonstrated that CAR-T cell-derived exosomes inhibit solid tumors, including triple negative breast cancer and lung cancer, and can affect the tumor microenvironment with relative safety (178, 179). Meanwhile, Haque et al. found that CAR-T cell-derived exosomes can be used to enhance the effects of cancer immunochemotherapy as well as to induce cell-contact toxicity (177, 180). These results demonstrate the great potential of T cell-derived exosomes for tumor therapy.

### 6.2 Natural killer cell

Natural killer cells (NK cells) are important immune cells in the body, derived from bone marrow, belong to the lymphocytes, the third type of lymphocytes except T cells and B cells, and can kill tumor cells non-specifically without prior sensitization (181). NK cells are classified into four main anti-tumor modalities: perforin/granzyme pathway, mediation of tumor apoptosis, ADCC pathway, and secretion of cytokines. NK cell-derived exosomes possess anti-tumor functions similar to those of their secreting cells (182). However, NK cells are a somewhat heterogeneous group of cells with differences in the function of their derived exosomes, potentially affecting future therapeutic outcomes. NK92 cells are human-derived NK cell lines that have a stable source, less difficulty in genetic modification manipulation relative to primary NK cells, and greater cytotoxicity and cytokine-producing capacity relative to primary NK cells (183). NK92 cells are human-derived NK cell lines with stable source, lower difficulty of gene modification operation relative to NK cells, and stronger cytotoxicity and cytokine-producing ability relative to NK cells, which is a hotspot for anti-tumor research at present (184). In view of the therapeutic properties of NK92 cell exosomes, researchers have modified the exosome surface proteins, combined them with single-chain antibodies recognizing tumor-associated antigens, and established the exosome surface display technology of single-chain antibodies to tumor-associated antigens, which enhances the broad-spectrum tumor targeting of the exosomes or the targeting of specific tumor cells or improves the therapeutic efficacy of antitumor drugs through the endogenous overexpression of effector molecules in the cells (185).

### 6.3 Other innate immune cells

Neutrophils are the most abundant innate immune cells circulating in the body, and their derived exosomes induce apoptosis in tumor cells by delivering cytotoxic proteins and activating the caspase signaling pathway (124). Based on these properties, the researchers modified N-Ex with superparamagnetic iron oxide nanoparticles (SPIONs) for higher tumor-targeting therapeutic effects. Meanwhile, exosome-loaded DOX was used to enhance the inhibition of tumor cells (124). Dendritic cells (DCs) are sentinel antigen-presenting cells of the immune system. DC-derived exosomes, which also contain functional MHC peptide complexes, co-stimulatory molecules, and other components that interact with immune cells, have the potential to promote immune cell-dependent tumor rejection and offer significant advantages over cell-based immunotherapies involving DC (186). Macrophages are immune cells that are widely distributed in the blood and tissues and are classified into M1 and M2 types (187, 188). M1-Exos)and M2-Exos have different functions, and the direction of macrophage polarization also affects the therapeutic efficacy, reprogramming of macrophages induces macrophage polarization in the M1-type direction and improves the therapeutic efficacy (189).

## 7 Conclusion

There is growing evidence that exosomes can be used as a vehicle for disease treatment with encouraging results. However, natural exosomes have some drawbacks, for example, heterogeneity, which can greatly reduce the effectiveness of disease treatment. Unlike natural exosomes, engineered exosomes can be modified in a variety of ways according to specific human wishes, thus enhancing or even conferring some new properties, and exosomes of immune cell origin have shown great potential in tumor therapy. However, the clinical translation of engineered exosomes also faces some difficulties, such as exosome isolation, quantification and analysis of exosomes in the clinical stage of engineered exosomes standardized methods still lack consensus, and how to accurately quantify the components of exosomes is also a difficult problem. Therefore, if we want to develop exosome-based drug delivery systems on a large scale in the clinic, we need to solve the above problems first.

## Author contributions

CS: Writing – original draft. JG: Writing – review & editing. XM: Writing – review & editing.

## Glossary

**Table:**

EV	extracellular vesicle
DOX	doxorubicin
MVB	multivesicular body
ESEs	early sorting endosomes
LSEs	late sorting endosomes
ILVs	intraluminal vesicles
EGFRvIII	epidermal growth factor receptor variant III
MSC-derived	Mesenchymal stem cell-derived
Lamp-2b	lysosome-associated membrane protein 2b
LA	lactomucin
GPI	glycosylphosphatidylinositol
CTP	cardiac-targeting peptide
AAV	Adeno-associated virus
LNPs	lipid nanoparticles
M1-exos	M1 - macrophage-derived exosomes
ASOs n	oligonucleotides
NOS2	itric oxide synthase 2
FAP	fibroblast activating protein alpha
CAF	cancer-associated fibroblasts
CTL	T-lymphocyte
CAR-T	constructing himeric antigen receptor T
NK cells	Natural killer cells
SPIONs	superparamagnetic iron oxide nanoparticles
PMN	pre-metastatic niche
GBM	glioblastoma
